# Evaluation of rebound tonometer iCare IC200 as compared with IcarePRO and Goldmann applanation tonometer in patients with glaucoma

**DOI:** 10.1186/s40662-021-00249-z

**Published:** 2021-07-01

**Authors:** Shunsuke Nakakura, Ryo Asaoka, Etsuko Terao, Yuki Nagata, Yasuko Fukuma, Satomi Oogi, Miku Shiraishi, Yoshiaki Kiuchi

**Affiliations:** 1 Department of Ophthalmology, Saneikai Tsukazaki Hospital, 68-1 Aboshi Waku, Himeji, 671-1227 Japan; 2 Department of Ophthalmology, Seirei Hamamatsu General Hospital, Hamamatsu, Shizuoka Japan; 3 Seirei Christopher University, Hamamatsu, Shizuoka Japan; 4 Department of Ophthalmology and Visual Sciences, Graduate School of Biomedical Sciences, Hiroshima University, Hiroshima, Japan

**Keywords:** Glaucoma, IC200, IcarePRO, Rebound tonometer, Goldmann applanation tonometer

## Abstract

**Background:**

This study investigated the agreement between a new rebound tonometer, IC200, and IcarePRO and Goldmann applanation tonometry (GAT).

**Methods:**

This was a prospective cross-sectional study. We measured the intraocular pressure (IOP) in 145 eyes of 145 glaucoma patients in the sitting position using GAT, IcarePRO, and IC200. IcarePRO and IC200 measurements were also obtained in the supine position. IC200 measurement was performed using two modes: single six (IC200-single) and automatic (IC200-continuous) six-measurements mode.

**Results:**

All tonometers provided high reproducibility in both positions (all intraclass correlation coefficients > 0.90), although it was highest with GAT, followed by IC200-continuous and IC200-single and then IcarePRO. In the sitting position, the mean (± SD) IOPs of GAT, IcarePRO, IC200-single, and IC200-continuous were 14.5 ± 2.9 mmHg, 13.3 ± 3.2 mmHg, 11.6 ± 3.2 mmHg, and 11.5 ± 3.2 mmHg, respectively. IOPs measured with IcarePRO or IC200 were significantly lower than those with GAT, particularly in patients with low IOP. IOPs measured with all tonometers were significantly elevated in the supine position as compared with the sitting position, but this difference was significantly greater with IC200-single and IC200-continuous compared with IcarePRO. IOP elevation was significant in eyes without bleb versus those with bleb, but this finding was not observed when IOP was measured with IcarePRO. The IOPs of the single and continuous modes of IC200 were interchangeable in both positions.

**Conclusions:**

GAT, IcarePRO, and IC200 had sufficiently high reproducibility, but measurements with IcarePRO may not be accurate in the supine position. Elevation of IOP in the supine position, especially in eyes with bleb, was more sensitively captured with IC200 than with IcarePRO.

**Trial registration:**

Japan Clinical Trials Register, No. UMIN000039982.

**Supplementary Information:**

The online version contains supplementary material available at 10.1186/s40662-021-00249-z.

## Background

Glaucoma is the second most common cause of blindness worldwide [1]. It is estimated that 79.6 million individuals will be living with glaucoma in 2020, and this number is likely to increase to 111.8 million in 2040 [2]. Because a 1-mmHg increase in intraocular pressure (IOP) increases the risk of the development glaucoma by 10 to 18% [3–5] and a 1-mmHg decrease in IOP reduces the progression of glaucoma by 10% [6], an accurate measurement of IOP is undoubtedly essential for glaucoma patients. Although the Goldmann applanation tonometer (GAT) has been the gold standard for the measurement of IOP, a series of rebound tonometers can measure IOP more objectively than other tonometers and without the use of topical anesthesia. These devices also enable the measurement of IOP even in bedridden patients, which is particularly important in patient populations with a growing life expectancy. In addition, the accurate measurement of IOP is especially important in pediatric glaucoma because it is typically performed on a bed with the patient under general anesthesia. The Icare tonometer series includes IcareTA01i, IcarePRO, IcareHOME, and IC100, but a direct measurement of IOP in the supine position is available only with IcarePRO. Moreover, among these rebound tonometers, the IOP measured with IcarePRO has the closest agreement with the IOP measured with GAT [7]. A new member has very recently joined the Icare series family i.e., IC200, which is expected to provide an even more accurate measurement of IOP, particularly in the supine position, because of its renewed position censor. Two recent reports have investigated the agreement between GAT and IC200, but these analyses were conducted only in the sitting position [8, 9]. The first aim of the current study is to compare the IOP values and their reproducibility measured with IC200, GAT, and IcarePRO in the sitting position. In addition, we measured IOP with IC200 and IcarePRO in the supine position and compared the IOP values and reproducibility. Furthermore, a secondary objective was to compare the conventional single measurement and continuous modes of IOP measurement, which can be used for automatically conducting six consecutive measurements by pushing the button only once. The second aim of the study was to investigate the effect of various ocular biomechanical parameters, such as central corneal thickness, on these measurements.

## Methods

This was a prospective cross-sectional study. Patients with glaucoma were recruited consecutively from the Department of Ophthalmology, Saneikai Tsukazaki Hospital, between May and August 2020. Inclusion criteria were 1) patients who suffered from glaucoma and had been visiting our clinic for longer than 3 months and 2) patients who agreed to the verbal explanation of the study and were able to lie on their back.

Exclusion criteria were patients who 1) were unable to undergo the GAT measurement, 2) had severe ocular surface condition 3) were unable to lie on a bed, 4) used contact lenses, and 5) did not agree to the verbal explanation of the research. This study was approved by the Institutional Review Board of Saneikai Tsukazaki Hospital (IRB No. 201012) and was performed according to the tenets of the Declaration of Helsinki. This clinical study was conducted as a part of a clinical trial registration (Japan Clinical Trials Register, No. UMIN000039982). Written informed consent was obtained from all participants before enrollment. We selected the patient’s eye with the worse visual field as measured with the Humphry Visual Field Analyzer (Carl Zeiss Inc., Dublin, CA) according to the SITA fast 24–2 program. If both eyes had similar visual field damage, the right eye was selected. Measurements of ocular biomechanical parameters were conducted before IOP measurements. Axial length, central corneal thickness, and corneal curvature (mean of horizontal and vertical) were measured using LENSTAR 900 (Haag-Streit, Koeniz, Switzerland) five times, and the mean value was used in the analyses.

### IOP measurements

#### GAT

A GAT measurement was conducted in all patients with their chins placed on the slit-lamp chin rest after administration of oxybuprocaine hydrochloride 0.4% (Benoxyl 0.4%, Santen Pharmaceutical Co., Ltd., Japan) with fluorescein. Before performing each test, the dial was set to approximately 15 mmHg. A single ophthalmologist (S.N.) performed the GAT measurement three times in all patients.

#### IcarePRO

**Fig. 1:**
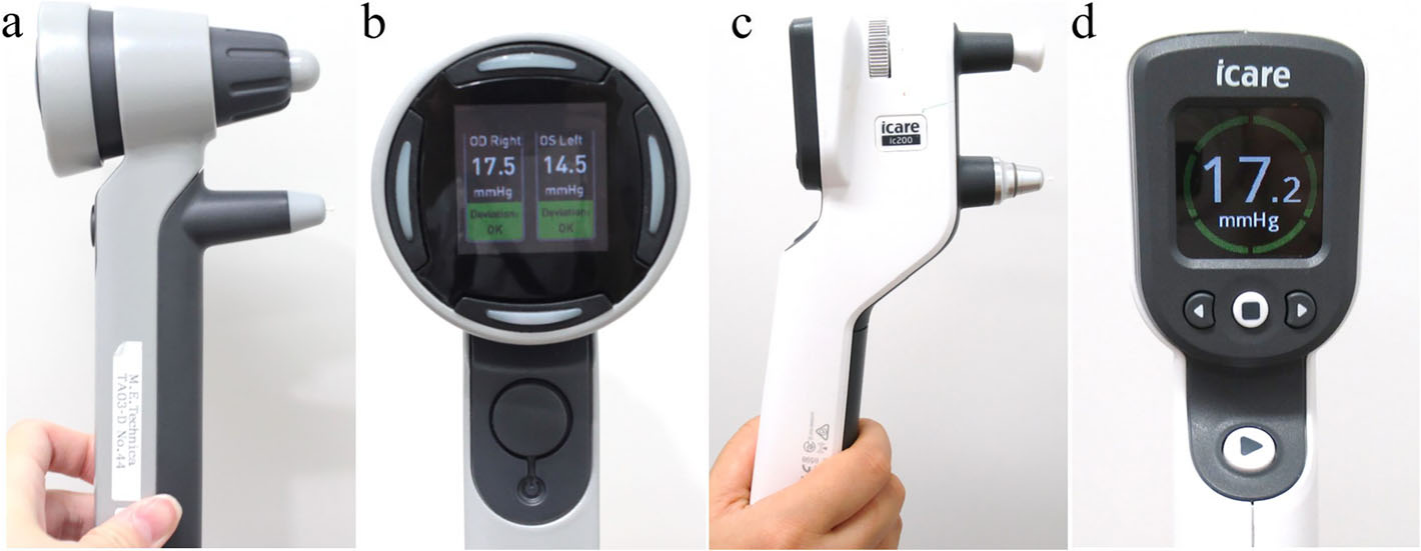
Rebound tonometers of IcarePRO and IC200. **a** Overview and **b** display of IcarePRO. **c** Overview and **d** display of the new rebound tonometer IC200

The IcarePRO (Fig. 1a; Icare Finland Oy, Helsinki, Finland) rebound tonometer has been available since 2011 and is the only Icare tonometer that allows for IOP measurement with the patient in the supine position. Similar to other earlier Icare tonometers, a probe is blasted on the center of the cornea at a distance between 3 mm and 7 mm, and the IOP is estimated by measuring the deceleration of the returning probe. However, IcarePRO uses a shorter probe than others do. After six consecutive measurements, the reliability derived from the variance of the measured values is displayed as the background color. Background colors of yellow or red indicate poor reliability, whereas green suggests a reliable measurement. We repeated the measurement until an IOP value with a green, yellow or red background appeared (Fig. 1b).

#### IC200

iCare IC200 (Icare Finland Oy; Fig. 1c, d) is an updated version of the IcarePRO (Fig. 1a, b) rebound tonometer. The mechanism of IOP measurement is very similar to the IcarePRO, but a long probe is used, as in IcareTA01i, IcareHOME, and IC100. With IC200, IOP can be measured even in the supine position with 200° of positional freedom. An improvement in IC200 as compared with IcarePRO is that its measurements are accepted only when performed correctly: perpendicularly from the center of the cornea at a distance of approximately 5 mm. IC200 has two measurement modes: a single measurement via a single push of the button (IC200-single) and automatic six-measurements measurement mode (IC200-continuous). Similar to the IcarePRO measurements, we recorded only IOP values on the green background (circle), which appeared after six consecutive measurements (Fig. 1d).

One of the five certified orthoptists (E.T., Y.N., Y.F., S.O., and M.S.) was randomized separately for each patient. The selected orthoptists measured the ocular biomechanical parameters, followed by IOP measurements with the patient in the sitting position. We first measured IOP using IcarePRO, IC200-single, and IC200-continuous in a randomized order. Each measurement was performed three times, and the mean value was calculated.

This was followed by the GAT measurement in the sitting position. Patients were then asked to lie flat in the supine position and in the same indoor brightness to the office for 10 min. Finally, IOP measurements using IcarePRO, IC200-single, and IC200-continuous were performed by the same selected orthoptist in a randomized order in the supine position. An interval of at least 1 min was given between each measurement. In some patients who had a narrow palpebral fissure, we used manual upper eyelid elevation when measuring IOP in the sitting position; however, this did not affect IOP values in Icare TA01i, Icare PRO, or Icare ic100 [10].

Each of the three IOP readings and the mean of these values were used in the between- and within-device data comparisons.

We conducted all measurements between 10:00 am and 2:00 pm.

### Statistical analysis

We evaluated the repeatability of three consecutive measurements for each tonometer by calculating the intraclass correlation coefficients (ICCs) as well as the coefficient of variance (CoV). According to the principles of McGraw and Wong, an ICC value greater than 0.7 is generally indicative of very good agreement [11]. IOP and CoV differences among the tonometer were analyzed using the Tukey’s multiple comparison test. The agreement among tonometers was evaluated using Bland–Altman analysis. Relationships between IOP values by GAT, IcarePRO, IC200-single, and IC200-continuous, as well as the values for age, axial length, corneal curvature, and central corneal thickness, were evaluated using multivariate linear regression. Nonpaired numerical values were compared using a nonpaired Wilcoxon signed-rank test.

Statistical analyses were performed using JMP version 10.0.0 (SAS Institute Inc., Cary, NC, USA) and statistical program R software (version 3.6.1, http://www.rproject.org/). Data were expressed as mean ± standard deviation (SD). *P* values less than 0.05 were considered statistically significant. We estimated the sample size to be 133 patients for detecting a 1 mmHg difference between the groups, with a significance level of 5% and a power of 80% based on an SD of 2.9 mmHg for the GAT by JMP.

## Results

### Patient demographics

Table 1 presents the demographic data of the studied 145 eyes of 145 patients (105 primary open-angle glaucoma patients, 19 exfoliation glaucoma, 12 normal tension glaucoma, 4 angle closure glaucoma, and 5 other glaucoma). The mean age of the patients was 65.6 ± 11.6 years (range, 35 to 86 years), of whom 82 (57%) were women. Of the eyes, 122 (84%) were right eyes, and 49 (34%) eyes underwent filtering surgery before the initiation of this study. Patients who had undergone a previous filtering surgery were regarded as having functional bleb; however, 7 patients (7/49) were treated with antiglaucoma medication.

**Table 1:** Patient demographics

Variable	Value
Eyes, R/L	122/23
Subjects, female/male	82/63
Age, mean ± SD (range), years	65.6 ± 11.6 (35 to 86)
Axial length, mean ± SD (range), mm	25.1 ± 1.8 (20.2 to 30.9)
Corneal curvature, mean ± SD (range), mm	7.61 ± 0.29 (6.89 to 8.45)
Central corneal thickness, mean ± SD (range), mm	0.522 ± 0.030 (0.412 to 0.606)

*SD* standard deviation

### Measurement in the sitting position (GAT, IcarePRO, IC200-single, and IC200-continuous)

#### IOP values

Table 2 shows IOP values in the sitting position. In this position, IOPs with GAT, IcarePRO, IC200-single, and IC200-continuous were 14.5 ± 2.9 (range, 5 to 22) mmHg, 13.3 ± 3.2 (6.3 to 23.6) mmHg, 11.6 ± 3.2 (3.6 to 21.2) mmHg, and 11.5 ± 3.2 (6.5 to 25.3) mmHg, respectively. A significant difference was found among the tonometers (*P* < 0.01, Tukey’s multiple comparison test), except for between IC200-single and IC200-continuous (*P* = 0.99).

**Table 2:** IO*P* value measured with each tonometer in the sitting and supine positions

Tonometer/position	Mean IOP, mean ± SD (range), mmHg
Sitting	
GAT	14.5 ± 2.9 (5 to 22)
IcarePRO	13.3 ± 3.2 (6.3 to 23.6)*
IC200-single	11.6 ± 3.2 (3.6 to 21.2)*^†^
I200-continuous	11.5 ± 3.2 (6.5 to 25.3)*^†^
Supine	
IcarePRO	14.5 ± 3.8 (6.5 to 25.3)
IC200-single	14.2 ± 4.4 (3.2 to 26.7)
IC200-continuous	14.3 ± 4.5 (3.4 to 29.5)

Tukey’s multiple-comparison test

*GAT* Goldmann applanation tonometry; *IOP* intraocular pressure; *SD* standard deviation

*P < 0.05 as compared with GAT

^†^*P* < 0.05 as compared with IcarePRO

#### Intradevice repeatability (ICC and CoV) (GAT, IcarePRO, IC200-single, and IC200-continuous)

The ICC values (> 0.90) suggested that intradevice repeatability was sufficiently high for all tonometers (Table 3), but IOP measured with GAT was associated with the highest ICC value, as suggested by the nonoverlapping 95% confidence interval (CI) of the ICC value with those of other devices. CoV values were superior with all tonometers, but the significantly smallest value was observed with GAT as compared with IcarePRO, IC200-single, and IC200-continuous in the sitting position (*P* < 0.01, Tukey’s multiple comparison test). IC200-continuous had a significantly smaller CoV value than IcarePRO (*P* < 0.05). There was no significant difference between the CoV values of IcarePRO and IC-200-single nor between IC200-continuous and IC200-single (*P* > 0.05).

**Table 3:** Intraclass coefficient and coefficient of variance of each intraocular pressure in the sitting and supine positions

Tonometer/position	ICC (95% CI)	CoV (%)
Sitting		
GAT	0.984 (0.979–0.988)	1.6 ± 2.2 (0.0–7.1)
IcarePRO	0.945 (0.929–0.959)	5.0 ± 3.2 (0.0–19.4)*
IC200-single	0.967 (0.957–0.975)	4.4 ± 2.2 (0.0–16.9)*
IC200-continuous	0.971 (0.962–0.978)	4.1 ± 2.8 (0.0–14.9)*^†^
Supine		
IcarePRO	0.963 (0.952–0.973)	4.6 ± 3.1 (0.0–17.2)
IC200-single	0.987 (0.984–0.991)	3.9 ± 2.2 (0.0–10.5)^†^
IC200-continuous	0.984 (0.979–0.984)	3.5 ± 2.3 (0.0–13.7)^†^

Tukey’s multiple-comparison test

*CI* confidence interval; *CoV* coefficient of variance; *ICC* intraclass correlation coefficient

*P < 0.05 as compared with GAT

^†^*P* < 0.05 as compared with IcarePRO

### Agreement between GAT and Icare measurements

Supplemental Table 1 shows all pair comparisons using Bland–Altman plots. All of the pair comparisons in the sitting position showed a relatively narrow width of 95% limit of agreement (LOA; 2.92–9.94 mmHg).

**Fig. 2:**
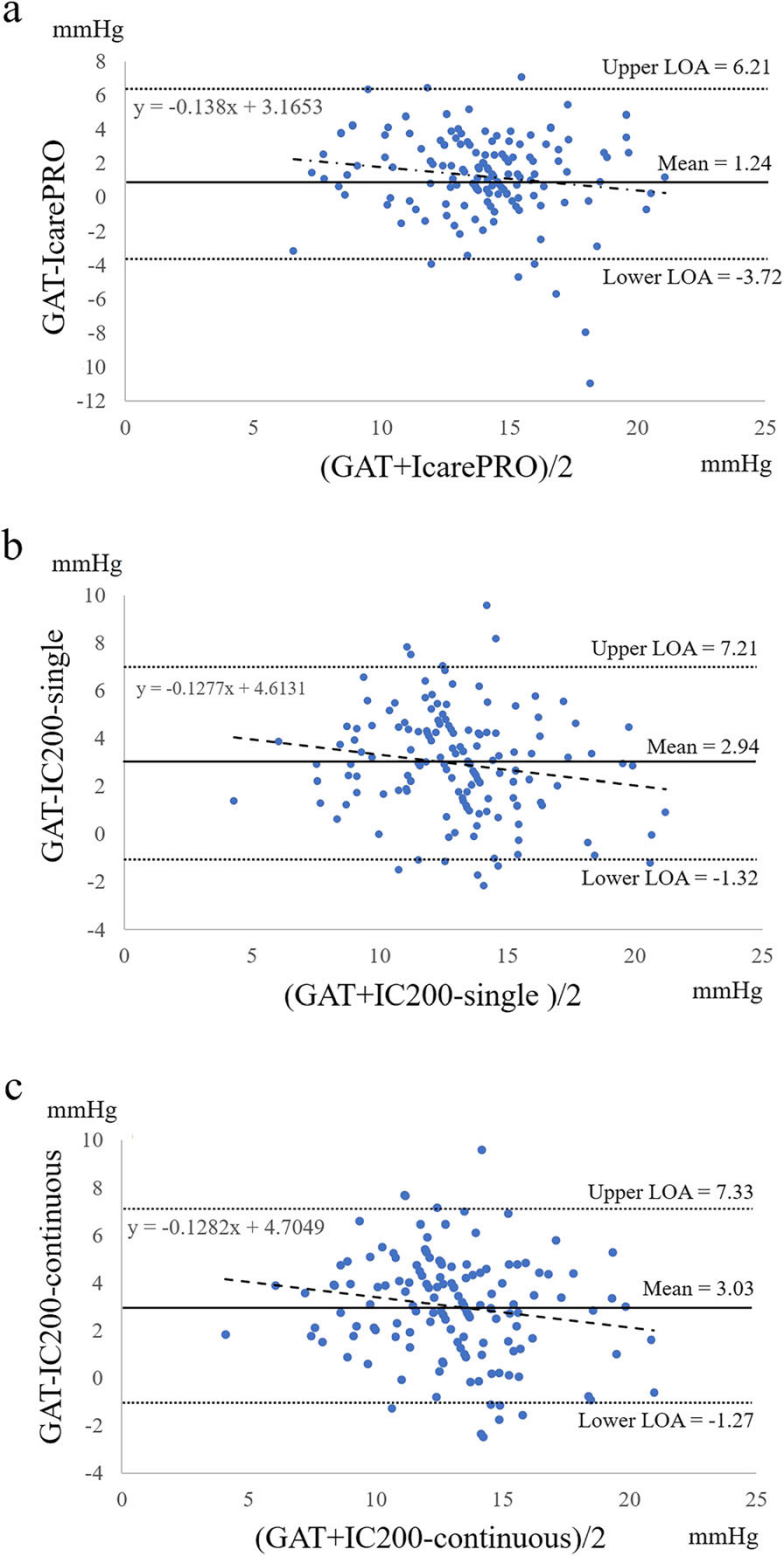
Bland–Altman plots of the measured IOPs between GAT and IcarePRO, between GAT and IC200-single, and between GAT and IC200-continuous at the sitting position. **a** GAT and IcarePRO, **b** GAT and IC200-single, **c** GAT and IC200-continuous. GAT, Goldmann applanation tonometry; LOA, limit of agreement; SD, standard deviation

Figure 2a shows the Bland–Altman plot between GAT and IcarePRO. The width of the 95% LOA was 9.93 mmHg. No significant trend was observed between the difference of the two IOPs and their means, although the *P* value was borderline (*r* = − 0.15, *P* = 0.066). Figure 2b presents the Bland–Altman plot between GAT and IC200-single. The width of the 95% LOA was 8.53 mmHg. We observed a significant trend in which IOP measured with IC200-single became smaller than GAT IOP when the average of these values was low (*r* = − 0.16, *P* < 0.05). The Bland–Altman plot between GAT and IC200-continuous is displayed in Fig. 2c. The width of the 95% LOA was 8.60 mmHg. There was a significant trend in which IOP measured with IC200-continuous became smaller than GAT IOP when the mean of these values was low (*r* = − 0.16, *P* < 0.05).

### Measurements in the supine position (IcarePRO, IC200-single, and IC200-continuous)

#### IOP values

Table 2 shows IOP values in the supine position. IOPs with IcarePRO, IC200-single, and IC200-continuous were 14.5 ± 3.8 (6.5 to 25.3) mmHg, 14.2 ± 4.4 (3.2 to 26.7) mmHg, and 14.3 ± 4.5 (3.4 to 29.5) mmHg, respectively. No significant difference was found among these values (*P* > 0.05, Tukey’s multiple comparison test).

#### Intradevice repeatability

As suggested by the ICC values (> 0.90), the intradevice repeatability of all Icare tonometers was sufficiently high (Table 3). CoV values were also sufficiently small among all Icare tonometers, but we observed a significantly smaller value with IC200-single and IC200-continuous as compared with IcarePRO (*P* < 0.01 and 0.05, respectively; Tukey’s multiple comparison test). There was no significant difference between IcarePRO and IC-200-single nor between IC200-continuous and IC200-single (*P* > 0.05).

### Agreement between IcarePRO and IC200 in the supine position

Supplemental Table 1 shows that in contrast to the sitting position, the agreement between IcarePRO IC-200-single or between IC200-continuous in the supine position became worse compared to those in the sitting position. The width of the 95% LOA was 16.87 mmHg and 17.06 mmHg, respectively.

### Agreement between IC200-single and IC200-continous in both positions

Supplemental Table 1 and Supplemental Figure 1 show that the difference in IOP between IC200-single and IC200-continous in sitting and supine positions was 0.09 mmHg and − 0.09 mmHg, respectively. In addition, the width of the 95% LOA was 2.92 mmHg and 3.36 mmHg, respectively. Thus, the value of the two measurement modes is thought to be interchangeable.

### Elevation of IOP in the supine position

**Fig. 3:**
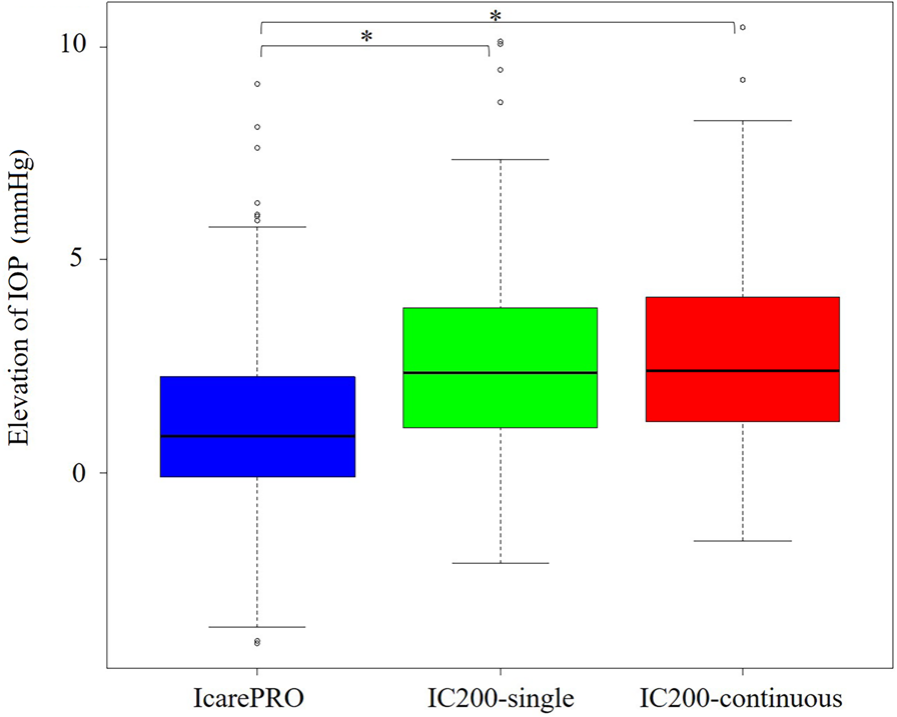
IOP elevation of IcarePRO, IC200-single, and IC200-continuous. The elevation of IOP was significantly different between IcarePRO and IC200. IcarePRO has less IOP elevation. *Significant pair by Tukey’s multiple comparison test

**Fig. 4:**
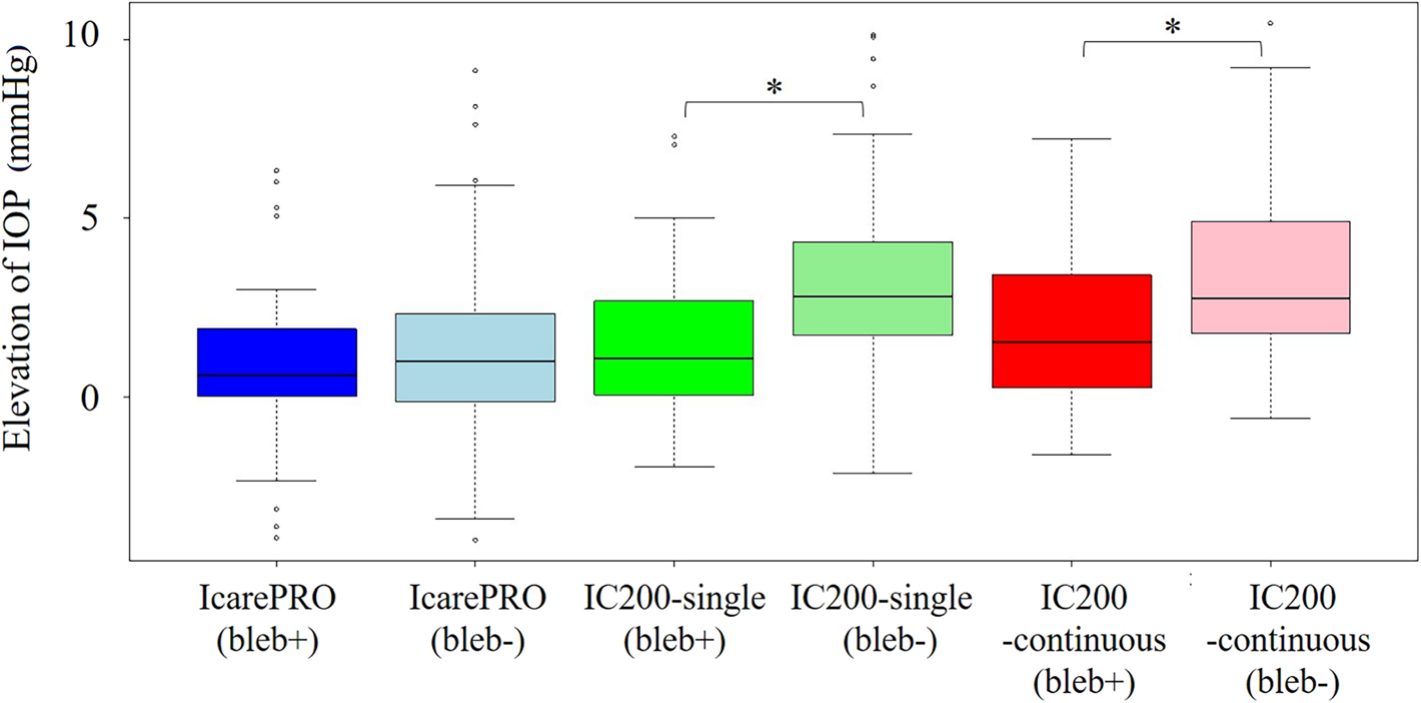
IOP elevation of each tonometer with and without bleb. The elevation of IOP was significantly different with and without bleb in IC200 but not in IcarePRO. *Significant pair by nonpaired Wilcoxon signed-rank test

IOP measurements of all Icare tonometers suggested that IOP was higher in the supine position as compared with the sitting position (*P* < 0.01, Tukey’s multiple comparison test), but this finding was significantly less obvious with IcarePRO (1.2 ± 2.2 [− 4.0 to 9.1] mmHg) than with IC200-single (2.8 ± 2.3 [− 1.6 to 10.5] mmHg) and IC200-continuous (2.6 ± 2.3 [− 2.1 to 10.2] mmHg; *P* < 0.01; Tukey’s multiple comparison test; see Fig. 3). As shown in Fig. 4, in eyes with bleb, the elevation of IOP in the supine position was 1.5 ± 2.1 (− 1.9 to 7.3) mmHg with IC200-single, which was significantly smaller (3.2 ± 2.0 [− 2.1 to 7.2] mmHg, *P* < 0.01, nonpaired Wilcoxon signed-rank test) than that in eyes without bleb. A similar tendency was observed with IC200-continuous (1.8 ± 2.0 [− 1.6 to 7.2] mmHg and 3.4 ± 2.2 [− 0.6 to 10.4] mmHg, *P* < 0.01). By contrast, there was no significant difference between these values with IcarePRO (0.9 ± 2.1 [− 0.6 to 6.3] mmHg and 1.4 ± 2.2 [− 4.0 to 9.2] mmHg, *P* = 0.29).

### Effects of age, axial length, corneal curvature, and central corneal thickness on IOP measurement with each tonometer

Table 4 presents the results of the multivariate linear regression analysis between each IOP and variables such as age, axial length, corneal curvature, and central corneal thickness. All analyses demonstrated that only central corneal thickness was correlated with IOP value (*P* < 0.01).

**Table 4:** Effects of age, axial length, corneal curvature, and central corneal thickness on IOP measurement with each tonometer^a^

Parameter	GAT		IcarePRO		IC200-single		IC200-continuous	
	Coefficient	*P* value	Coefficient	*P* value	Coefficient	*P* value	Coefficient	*P* value
Sitting								
Age	0.022	0.34	−0.022	0.39	−0.0028	0.91	0.029	0.91
Central corneal thickness	**0.024**	**0.0021**	**0.028**	**0.0012**	**0.028**	**0.0011**	**0.031**	**0.00041**
Corneal curvature	0.29	0.73	0.15	0.87	1.10	0.19	1.21	0.19
Axial length	0.0031	0.98	0.20	0.21	0.13	0.42	0.13	0.41
Supine								
Age	–	–	0.016	0.60	0.0011	0.97	0.015	0.67
Central corneal thickness	–	–	**0.025**	**0.017**	**0.031**	**0.014**	**0.032**	**0.0097**
Corneal curvature	–	–	−0.29	0.79	1.21	0.36	1.38	0.30
Axial length	–	–	0.28	0.15	0.072	0.75	0.16	0.48

*IOP* intraocular pressure

^a^Boldface type indicates *P* < 0.05

The results of the correlation coefficient calculations (*r* and *P* values) between IOP measurements as taken by the GAT, IcarePRO, IC200-single, and IC200-continuous and age, axial length, corneal curvature, and central corneal thickness are shown in Supplemental Table 2. All tonometer values were affected by the central corneal thickness (all *r* > 0.20, all *P* < 0.05).

## Discussion

In the current study, IOP was measured using GAT, IcarePRO, IC200-single, and IC200-continuous in 145 eyes of 145 patients with glaucoma. The measurements with IcarePRO, IC200-single, and IC200-continuous were also conducted in the supine position. As a result, all tonometric measurements had sufficiently high reproducibility, although it was highest with GAT, followed by IC200-single and IC200-continuous and lastly, IcarePRO. All mean IOP values measured with IcarePRO, IC200-single, and IC200-continuous were lower as compared with GAT IOP (Table 2) and GAT IOP tended to be higher than IOPs with Icares, particularly when IOP was low (Supplemental Table 1). The elevation of IOP in the supine position was observed with all Icares, but it was more obvious with IC200-single and IC200-continuous than with IcarePRO. Moreover, we found that this IOP elevation was more obvious in eyes without bleb than in those with bleb when measured with IC200-single and IC200-continuous, but this phenomenon was not observed when IcarePRO was used.

In the current study, IOP measured with IcarePRO was lower than that measured by GAT (by 1.24 mmHg in the sitting position). This difference was within the range of the repeatability of GAT (between 2.2 mmHg and 2.9 mmHg) [12–15] but was larger than those in other previous studies (between 0.01 mmHg and 0.43 mmHg) [16–20]. In the Bland–Altman plot, the LOA was 9.93 mmHg, which was also marginally larger than that reported in previous studies (i.e., between 5.2 mmHg and 8.94 mmHg) [16–20]. The reason for these disagreements is unclear but could be attributed to the difference in the very detailed measurement method of IcarePRO in each study because IcarePRO is largely influenced by manual eyelid manipulation [16]. As for IC200, in this study, the mean IOP was approximately 3 mmHg lower than that of GAT IOP. Earlier reports that compared IC200 and GAT indicated scattered results [8, 9]. Badakere et al. [8]. reported that the IOP of IC200 was higher than that of GAT by 1.27 mmHg in 96 patients with glaucoma and 60 healthy subjects. Perez-Garcia et al. [9]. also reported that IC200 was higher than GAT by 0.82 mmHg in 40 patients with congenital glaucoma and 42 healthy subjects. In the current study, the LOA in the Bland–Altman plot was 8.53 or 8.60 mmHg, which was considerably larger than the value reported in previous studies (i.e., 4.3 mmHg) [8]. The apparent cause of these differences is uncertain, but one of the reasons could be the difference in the patients’ backgrounds, including ethnicity, whether or not the patient had undergone filtering surgery, whether they were healthy subjects or had glaucoma, as well as the difference in ocular biomechanical properties.

All IOP values in the current study measured by GAT, IcarePRO, and IC200 were affected by the central corneal thickness (Table 4; Supplemental Table 2; all *r* > 0.20, *P* < 0.05), which agrees with the findings of previous studies using IC200 (*r* = 0.32) [9], GAT (*r* = 0.16-0.28) [15, 20], and IcarePRO (*r* = 0.26) [20]. Similar findings have also been reported with other Icare tonometers, including IcareHOME (*r* = 0.40) [21], IC100 (*r* = 0.50) [22], and IcareTA01i (*r* = 0.46) [22]. The effect of corneal curvature on IOP values with rebound tonometers has also been reported: IcareTA01i (*r* = 0.27) [23] and IC100 (*r* = 0.25) [22]. Age has also been shown to be associated with Icare IOPs: IcareTA01i (*r* = − 0.38) [22] and IC100 (*r* = − 0.37) [22]. These associations were not observed in the current study (Table 4; Supplemental Table 2). No studies have reported the effect of axial length on IOP values of rebound tonometers.

IOP changes between the sitting and supine positions were different between IcarePRO and IC200. IOP did not change in IcarePRO but apparently increased in IC200. Our previous study using IcarePRO among 127 healthy subjects showed the same tendency in IcarePRO, i.e., no significant difference in IOP between the sitting and supine positions (15.5 vs. 15.8 mmHg, respectively, *P* = 0.301 by Mann–Whitney U test) [19].

A few previous reports have indicated that the increase in IOP in the supine position is reduced after filtering surgery. Hirooka et al. [23]. measured IOP in both the sitting and supine positions using a pneumatonometer. They reported that the increase in IOP in the supine position was, on average, 4.1 mmHg before trabeculectomy and 2.2 mmHg after trabeculectomy [23]. Then, Sawada and Yamamoto [24] reported that the increase in IOP in the decubitus position was 3.9 mmHg in eyes without bleb and 1.3 mmHg in eyes with bleb when IOP was measured with the IcareTA01i [24]. In this study, increased IOP was also significantly lower in eyes with blebs (1.5 mmHg and 1.8 mmHg) using IC200-single and IC200-continuous than that in eyes without bleb (3.2 and 3.4 mmHg, respectively). However, increased IOP as measured by IcarePRO was not significantly different from that in eyes with bleb (0.9 mmHg) and in eyes without bleb (1.4 mmHg). We believe that increased IOP in the supine position was more sensitively captured using IC200 than using IcarePRO. Thus, IcarePRO may not be suitable for measuring IOP in the supine position because of its low reproducibility and less sensitivity in capturing measurements for increase in IOP.

Finally, with regard to the two measurement modes, IC200-single and IC200-continuous, no significant difference was observed between the measured IOPs in either the sitting or supine position (Table 2; Supplemental Table 1). In both positions, the difference between two measurement methods was approximately 0 mmHg, and the width of the 95% LOA was only approximately 3 mmHg (Supplemental Figure 1). Therefore, both measurement methods provide completely high agreement and can be considered to be interchangeable. This exaggerates the merit of IC200-continuous because of the much shorter measurement time compared with IC200-single (7.3 s with IC200-single and 2.2 s with IC200-continuous over five consecutive measurements in a part of the current study).

A limitation of our study was that all our subjects were older glaucoma patients. Therefore, our results might not be directly applicable to healthy subjects or children. In addition, patients with glaucoma have different biomechanical properties as compared with healthy subjects [25], which will affect the difference in IOP among tonometers and increase the difference as compared with that of previous studies. The second limitation of this study was that we did not include eyes with extremely high IOP, i.e., > 30 mmHg, and thus further studies are needed to shed light on this aspect. The third limitation was the use of a single person to perform tonometry for GAT, which will increase the repeatability, and we did not measure IOP with GAT in the supine position.

## Conclusions

The new position-independent rebound tonometer, IC200, provides high reproducibility in both the sitting and supine positions. In general, there was good agreement among GAT, IC200, and IcarePRO, but the elevation of IOP in the supine position, especially in eyes with bleb, was more sensitively captured with IC200 than with IcarePRO. IOPs of the single and continuous modes of IC200 were interchangeable. Our results will be useful for clinical practice and glaucoma management.

## Supplementary Information

**Additional file 1: Supplemental Figure 1.** Bland–Altman plots of measured IOPs between IC200-single and IC200-continuous in sitting and supine positions. **Supplemental Table 1.** Results of Bland–Altman analysis and Pearson’s correlation coefficient tests. **Supplemental Table 2.** Results of correlation coefficient calculations (r and *P* values) between IOP measurements as taken by GAT, IcarePRO, IC200-single, and IC200-continuousa.

## Data Availability

The datasets used in the current study are available from the corresponding author (SN) upon reasonable request.

## Abbreviations

IOP: Intraocular pressure
GAT: Goldmann applanation tonometer
ICCs: Intraclass correlation coefficients
CoV: Coefficient of variance
SD: Standard deviation
LOA: Limit of agreement

## References

[1] Available at https://www.worldglaucomaweek.org/what-is-glaucoma/. Accessed 23 Oct 2020.

[2] Available at https://www.glaucomapatients.org/basic/statistics/. Accessed 23 Oct 2020.

[3] Bengtsson B, Heijl A. A long-term prospective study of risk factors for glaucomatous visual field loss in patients with ocular hypertension. J Glaucoma. 2005;14(2):135–8. 10.1097/01.ijg.0000151683.04410.f3 15741815

[4] Leske MC, Wu SY, Hennis A, Honkanen R, Nemesure B, BESs Study Group. Risk factors for incident open-angle glaucoma: the Barbados Eye Studies. Ophthalmology. 2008;115(1):85–9. 10.1016/j.ophtha.2007.03.017 17629563

[5] de Voogd S, Ikram MK, Wolfs RC, Jansonius NM, Hofman A, de Jong PT. Incidence of open-angle glaucoma in a general elderly population: the Rotterdam Study. Ophthalmology. 2005;112(9):1487–93. 10.1016/j.ophtha.2005.04.018 16039716

[6] Leske MC, Heijl A, Hussein M, Bengtsson B, Hyman L. Komaroff E; Early Manifest Glaucoma Trial Group. Factors for glaucoma progression and the effect of treatment: the early manifest glaucoma trial. Arch Ophthalmol. 2003;121(1):48–56. 10.1001/archopht.121.1.48 12523884

[7] Nakakura S. Icare® rebound tonometers: review of their characteristics and ease of use. Clin Ophthalmol. 2018;12:1245–53. 10.2147/OPTH.S163092 PMC6047858 30034218

[8] Badakere SV, Chary R, Choudhari NS, Rao HL, Garudadri C, Senthil S. Agreement of intraocular pressure measurement of Icare ic200 with Goldmann applanation tonometer in adult eyes with normal cornea. Ophthalmol Glaucoma. 2021;4(1):89–94. 10.1016/j.ogla.2020.08.004 32801019

[9] Perez-Garcia P, Morales-Fernandez L, Saenz-Frances F, Mendez-Hernandez CD, Garcia-Feijoo J, Santos-Bueso E, et al. Comparision of intraocular pressure measured using the new Icare 200™ rebound tonometer and the Perkins™ applanation tonometer in healthy subjects and in patients with primary congenital glaucoma. Arch Soc Esp Oftalmol (Engl Ed). 2021;96(4):175–80. 10.1016/j.oftal.2020.06.007 32690372

[10] Nakakura S, Mori E, Fujio Y, Fujisawa Y, Matsuya K, Kobayashi Y, et al. Effect of manual upper eyelid elevation on intraocular pressure measurement by four different tonometers. Optom Vis Sci. 2020;97(2):128–33. 10.1097/OPX.0000000000001472 32011586

[11] Tonnu PA, Ho T, Sharma K, White E, Bunce C, Garway-Heath D. A comparison of four methods of tonometry: method agreement and interobserver variability. Br J Ophthalmol. 2005;89(7):847–50. 10.1136/bjo.2004.056614 PMC1772716 15965164

[12] Dielemans I, Vingerling JR, Hofman A, Grobbee DE, de Jong PT. Reliability of intraocular pressure measurement with the Goldmann applanation tonometer in epidemiological studies. Graefes Arch Clin Exp Ophthalmol. 1994;232(3):141–4. 10.1007/BF00176782 8188062

[13] Pandav SS, Sharma A, Gupta A, Sharma SK, Gupta A, Patnaik B. Reliability of ProTon and Goldmann applanation tonometers in normal and postkeratoplasty eyes. Ophthalmology. 2002;109(5):979–84. 10.1016/s0161-6420(02)00974-0 11986107

[14] Kotecha A, White E, Schlottmann PG, Garway-Heath DF. Intraocular pressure measurement precision with the Goldmann applanation, dynamic contour, and ocular response analyzer tonometers. Ophthalmology. 2010;117(4):730–7. 10.1016/j.ophtha.2009.09.020 20122737

[15] Güler M, Bilak Ş, Bilgin B, Şimşek A, Çapkin M, Hakim Reyhan A. Comparison of intraocular pressure measurements obtained by Icare PRO rebound tonometer, Tomey FT-1000 noncontact tonometer, and Goldmann applanation tonometer in healthy subjects. J Glaucoma. 2015;24(8):613–8. 10.1097/IJG.0000000000000132 25264986

[16] Baek SU, Ha A, Kim YK, Jeoung JW, Park KH. Effect of manual eyelid manipulation on intraocular pressure measurement by rebound tonometry. Br J Ophthalmol. 2018;102(11):1515–9. 10.1136/bjophthalmol-2017-311587 29420194

[17] Moreno-Montañés J, Martínez-de-la-Casa JM, Sabater AL, Morales-Fernandez L, Sáenz C, Garcia-Feijoo J. Clinical evaluation of the new rebound tonometers Icare PRO and Icare ONE compared with the Goldmann tonometer. J Glaucoma. 2015;24(7):527–32. 10.1097/IJG.0000000000000058 24844537

[18] Jablonski KS, Rosentreter A, Gaki S, Lappas A, Dietlein TS. Clinical use of a new position-independent rebound tonometer. J Glaucoma. 2013;22(9):763–7. 10.1097/IJG.0b013e318259aa47 23172572

[19] Nakakura S, Mori E, Yamamoto M, Tsushima Y, Tabuchi H, Kiuchi Y. Intradevice and interdevice agreement between a rebound tonometer, Icare PRO, and the Tonopen XL and Kowa hand-held applanation tonometer when used in the sitting and supine position. J Glaucoma. 2015;24(7):515–21. 10.1097/IJG.0000000000000016 24145289

[20] Kato Y, Nakakura S, Matsuo N, Yoshitomi K, Handa M, Tabuchi H, et al. Agreement among Goldmann applanation tonometer, iCare, and Icare PRO rebound tonometers; non-contact tonometer; and Tonopen XL in healthy elderly subjects. Int Ophthalmol. 2018;38(2):687–96. 10.1007/s10792-017-0518-2 28393323

[21] Takagi D, Sawada A, Yamamoto T. Evaluation of a new rebound self-tonometer, Icare HOME: comparison with Goldmann applanation tonometer. J Glaucoma. 2017;26(7):613–8. 10.1097/IJG.0000000000000674 28369004

[22] Nakakura S, Mori E, Fujio Y, Fujisawa Y, Matsuya K, Kobayashi Y, et al. Comparison of the intraocular pressure measured using the new rebound tonometer Icare ic100 and Icare TA01i or Goldmann applanation tonometer. J Glaucoma. 2019;28(2):172–7. 10.1097/IJG.0000000000001138 30689609

[23] Hirooka K, Takenaka H, Baba T, Takagishi M, Mizote M, Shiraga F. Effect of trabeculectomy on intraocular pressure fluctuation with postural change in eyes with open-angle glaucoma. J Glaucoma. 2009;18(9):689–91. 10.1097/IJG.0b013e31819c49f4 20010249

[24] Sawada A, Yamamoto T. Effects of trabeculectomy on posture-induced intraocular pressure changes over time. Graefes Arch Clin Exp Ophthalmol. 2012;250(9):1361–6. 10.1007/s00417-012-1942-7 22323246

[25] Wang W, Du S, Zhang X. Corneal deformation response in patients with primary open-angle glaucoma and in healthy subjects analyzed by Corvis ST. Invest Ophthalmol Vis Sci. 2015;56(9):5557–65. 10.1167/iovs.15-16926 26305527

