# Supplementary material for: Evaluation of rebound tonometer iCare IC200 as compared with IcarePRO and Goldmann applanation tonometer in patients with glaucoma
Source: Eye Vis (Lond). 2021 Jul 1;8:25. doi: 10.1186/s40662-021-00249-z (PMC8247177; doi:10.1186/s40662-021-00249-z)
Supplement: Supplementary file 1 — Additional file 1: Supplemental Figure 1. Bland–Altman plots of measured IOPs between IC200-single and IC200-continuous in sitting and supine positions. Supplemental Table 1. Results of Bland–Altman analysis and Pearson’s correlation coefficient tests. Supplemental Table 2. Results of correlation coefficient calculations (r and P values) between IOP measurements as taken by GAT, IcarePRO, IC200-single, and IC200-continuousa. [file 40662_2021_249_MOESM1_ESM.zip › Supplemental material.docx]

Supplemental FIGURE1

Bland–Altman plots of measured IOPs between IC200-single and IC200-continuous in sitting and supine positions.

a. sitting position, b. supine position. LOA, limit of agreement; SD, standard deviation.

**Supplemental Table 1. Results of Bland–Altman analysis and Pearson’s correlation coefficient tests**

|  | Correlation coefficient | |  | Difference between 2 measurements (mmHg) | |  | LOA (mmHg) | | |
| --- | --- | --- | --- | --- | --- | --- | --- | --- | --- |
| Bland–Altman analysis | r | *P* value |  | Mean | SD |  | Lower 95% | Upper 95% | Width of 95% |
| Sitting position |  |  |  |  |  |  |  |  |  |
| GAT and IcarePRO | −0.15 | 0.06 |  | 1.24 | 2.53 |  | −3.72 | 6.21 | 9.94 |
| GAT and IC200-single | −0.16 | <0.05 |  | 2.94 | 2.17 |  | −1.32 | 7.21 | 8.54 |
| GAT and IC200-continuous | −0.16 | <0.05 |  | 3.03 | 2.19 |  | −1.27 | 7.33 | 8.61 |
| IcarePRO and IC200-single | 0.01 | <0.001 |  | 1.70 | 1.70 |  | −1.64 | 5.04 | 6.69 |
| IcarePRO and IC200-continuous | 0.01 | <0.001 |  | 1.79 | 1.72 |  | −1.58 | 5.16 | 6.75 |
| IC200-single and IC200-continuous | −0.00 | 0.598 |  | 0.09 | 0.74 |  | −1.30 | 1.55 | 2.92 |
| Supine position |  |  |  |  |  |  |  |  |  |
| IcarePRO and IC200-single | −0.66 | <0.05 |  | 3.17 | 4.30 |  | −5.26 | 11.61 | 16.87 |
| IcarePRO and IC200-continuous | −0.67 | <0.05 |  | 3.07 | 4.35 |  | −5.45 | 11.60 | 17.06 |
| IC200-single and IC200-continuous | −0.05 | <0.05 |  | −0.09 | 0.85 |  | −1.77 | 1.58 | 3.36 |

Correlation coefficient *r* and *P* value indicate the relationship between A − B and (A+B)/2 in the Bland–Altman analysis.

Abbreviation: LOA, limits of agreements calculated by mean difference ± standard deviation

**Supplemental Table 2.** Results of correlation coefficient calculations (*r* and *P* values) between IOP measurements as taken by GAT, IcarePRO, IC200-single, and IC200-continuous^a^

|  | Correlation (*r*) (95% CI) | | | |
| --- | --- | --- | --- | --- |
| Tonometer/position | Age | Axial length | Corneal curvature | Central corneal thickness |
| Sitting |  |  |  |  |
| GAT | 0.07 (−0.09 to 0.23) | −0.01 (−0.17 to 0.15) | 0.03 (−0.12 to 0.20) | **0.25 (0.09–0.40)** |
| IcarePRO | −0.14 (−0.29 to 0.02) | 0.16 (0.00 to 0.32) | 0.07 (−0.08 to 0.23) | **0.27 (0.12–0.42)** |
| IC200-single | −0.07 (−0.23 to 0.09) | 0.11 (−0.04 to 0.27) | 0.14 (−0.02 to 0.29) | **0.28 (0.12–0.42)** |
| IC200-continuous | −0.05 (−0.25 to 0.10) | 0.11 (−0.05 to 0.10) | 0.15 (−0.01 to 0.30) | **0.30 (0.14–0.44)** |
| Supine |  |  |  |  |
| IcarePRO | −0.01 (−0.17 to 0.14) | 0.12 (−0.04 to 0.27) | 0.01 (−0.14 to 0.17) | **0.20 (0.04–0.35)** |
| IC200-single | −0.03 (−0.19 to 0.13) | 0.05 (−0.10 to 0.21) | 0.10 (−0.05 to 0.26) | **0.21 (0.05–0.36)** |
| IC200-continuous | −0.01 (−0.17 to 0.14) | 0.08 (−0.08 to 0.24) | 0.11 (−0.04 to 0.27) | **0.22 (0.06–0.37)** |

^a^Boldface type indicates statistically and clinically significant values (*P*<0.05). All tonometers were affected by central corneal thickness.
