# Supplementary figures and images for: Evaluation of rebound tonometer iCare IC200 as compared with IcarePRO and Goldmann applanation tonometer in patients with glaucoma
Source: Eye Vis (Lond). 2021 Jul 1;8:25. doi: 10.1186/s40662-021-00249-z (PMC8247177; doi:10.1186/s40662-021-00249-z)

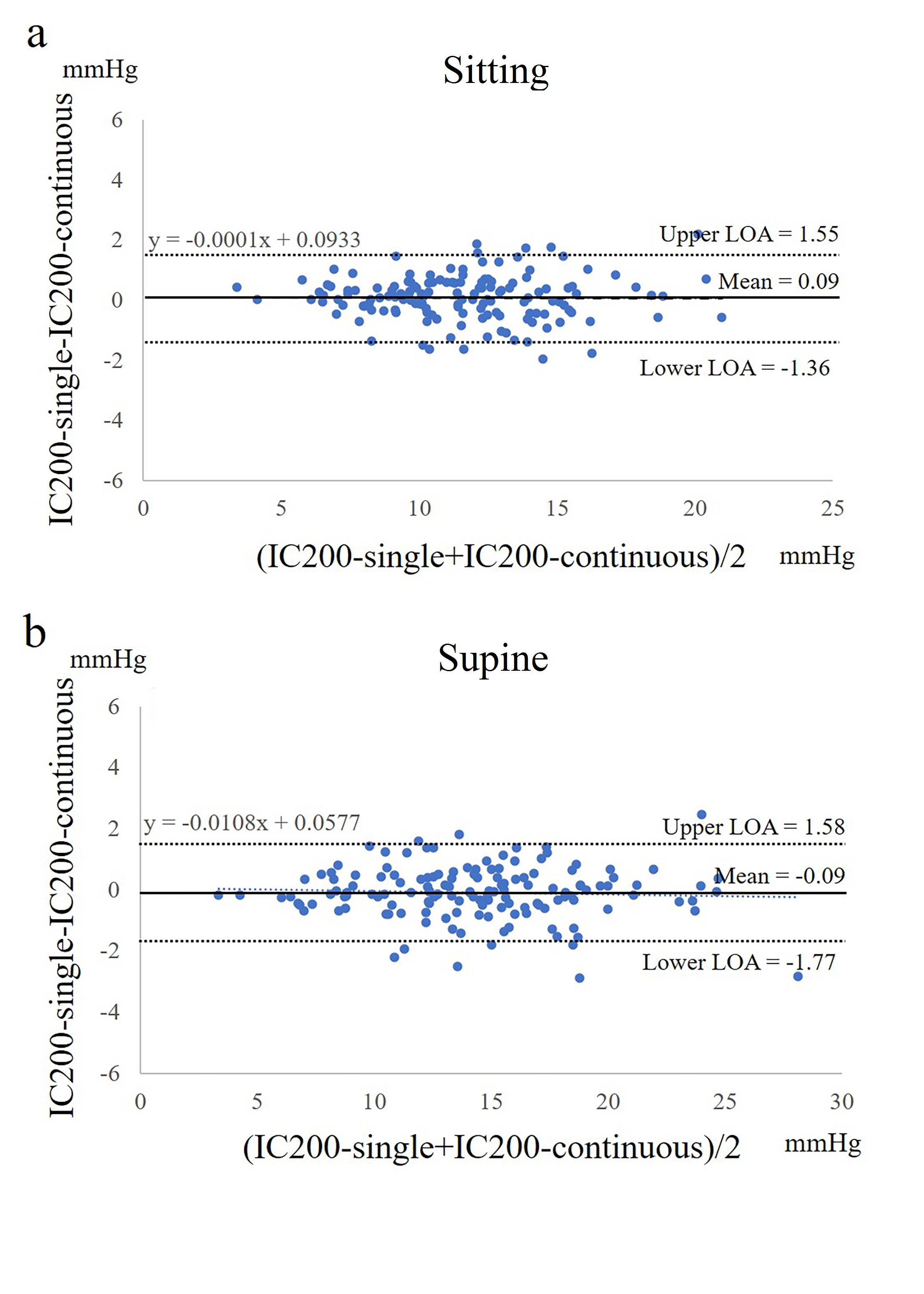

Supplement: Supplementary file 1 — Additional file 1: Supplemental Figure 1. Bland–Altman plots of measured IOPs between IC200-single and IC200-continuous in sitting and supine positions. Supplemental Table 1. Results of Bland–Altman analysis and Pearson’s correlation coefficient tests. Supplemental Table 2. Results of correlation coefficient calculations (r and P values) between IOP measurements as taken by GAT, IcarePRO, IC200-single, and IC200-continuousa. [file 40662_2021_249_MOESM1_ESM.zip › Suppl Figure 01.tif]
